# A Novel Neural Network Model Based on Real Mountain Road Data for Driver Fatigue Detection

**DOI:** 10.3390/biomimetics10020104

**Published:** 2025-02-12

**Authors:** Dabing Peng, Junfeng Cai, Lu Zheng, Minghong Li, Ling Nie, Zuojin Li

**Affiliations:** School of Electronic and Electrical Engineering, Chongqing University of Science and Technology, Chongqing 401331, China; 2023204020@cqust.edu.cn (D.P.); 2023204026@cqust.edu.cn (J.C.); 2023204023@c-qust.edu.cn (L.Z.); 2022204024@cqust.edu.cn (M.L.); 2005050@cqust.edu.cn (L.N.)

**Keywords:** fatigue driving detection, YOLOv5, TA mechanism, feature extraction

## Abstract

Mountainous roads are severely affected by environmental factors such as insufficient lighting and shadows from tree branches, which complicates the detection of drivers’ facial features and the determination of fatigue states. An improved method for recognizing driver fatigue states on mountainous roads using the YOLOv5 neural network is proposed. Initially, modules from Deformable Convolutional Networks (DCNs) are integrated into the feature extraction stage of the YOLOv5 framework to improve the model’s flexibility in recognizing facial characteristics and handling postural changes. Subsequently, a Triplet Attention (TA) mechanism is embedded within the YOLOv5 network to bolster image noise suppression and improve the network’s robustness in recognition. Finally, the Wing loss function is introduced into the YOLOv5 model to heighten the sensitivity to micro-features and enhance the network’s capability to capture details. Experimental results demonstrate that the modified YOLOv5 neural network achieves an average accuracy rate of 85% in recognizing driver fatigue states.

## 1. Introduction

Throughout the ‘14th Five-Year Plan’ phase, the State Council and other regulatory authorities issued a series of policies such as the ‘14th Five-Year Plan for Advancing a Modern Comprehensive Transportation Network’, concentrating on the expansion of automatic driving application scenarios and the development of intelligent driving technology. Fatigue driving detection is an important direction in the field of intelligent driving, and driver fatigue detection under the condition of mountain roads is a difficult point, because mountain roads are seriously affected by environmental factors such as insufficient light, tree shadow, special scene light (dusk, night), and so on, making fatigue determination based on facial features more complicated.

At present, traffic accidents are still one of the major life-threatening hazards. Lack of road safety awareness, drinking and driving, and fatigued driving are all key traffic safety hazards. The proportion of traffic accidents caused by fatigued drivers ranges between 14% and 20%, while the proportion of serious traffic accidents is about 43% [1,2]. The dangers of fatigued driving should not be underestimated, as it can significantly slow down a driver’s reaction time and judgment, thus increasing the likelihood of an accident. Consequently, the challenge of efficiently detecting and mitigating fatigue driving has emerged as a crucial topic in transportation safety studies [3,4].

Currently, research on fatigue driving focuses on the following aspects: fatigue detection based on physiological signals [5,6], fatigue detection based on driving behavior [7,8], fatigue detection based on computer vision [9,10], and multimodal fusion detection methods.

Methods for detecting physiological signals primarily focus on tracking the driver’s brain activity (EEG), heart rate (HR), and galvanic skin response (GSR), from which specific indicators related to fatigue are derived [11,12,13]. Zhendong Mu et al. [14] collected EMG and respiratory signals from drivers through sensors and processed the signals using an approximate entropy method to detect fatigue using a set threshold. Meanwhile, Jaewon Lee et al. [15] used multi-information fusion technology to identify the driver’s fatigue state using a convolutional neural network algorithm using four signals, namely, EEG, ECG, EMG, and oximetry, as the data source, and obtained high detection accuracy and reliability. However, this type of method requires drivers to wear multiple sensors, which can seriously affect driving behavior, and thus is difficult to be applied on a large scale.

Driving behavior detection methods, on the other hand, identify fatigue by analyzing vehicle driving data such as speed, steering angle [16,17], and acceleration [18,19]. Zhao et al. [20] proposed a driver fatigue detection method using EEG signals and a hybrid CNN-LSTM deep learning model, achieving an accuracy of 92.3%, outperforming traditional methods. In their research, Le Xu, Huixing Zhou, and colleagues [21] employed an enhanced convolutional neural network (CNN) model to detect driver fatigue, utilizing data from steering wheel turn angles and traverse angle measurements. The method showed high detection performance by extracting complex entropy features of the signals and verified under real driving conditions, with a recognition accuracy of 87.6%.

The application of computer vision technology provides new ideas for fatigue driving detection, especially for recognizing fatigue by monitoring the driver’s facial features (e.g., the state of the eyes and mouth). Studies have shown that facial features such as drivers’ eye blinking frequency, eyelid closure time, and mouth opening frequency are closely related to fatigue status [22]. These features can be detected in real time by cameras and image processing algorithms to enable early warning of fatigued driving.

Determination of driver fatigue features [23] can be divided into three main stages by analyzing the face state: face localization, feature extraction and fatigue state determination. Before the popularization of deep learning applications, researchers such as VIOLA [24] have used the so-called “whole-image” technique to capture image features and combined it with the AdaBoost classifier through a “cascade” approach to efficiently achieve face recognition. Jian Chen et al. used the AdaBoost method for identifying faces and tracked face motion with the Kalman filter algorithm. Then, 68 facial marker points were detected using a cascade regression tree to compute and derive the eye aspect ratio more precisely. An advanced BP neural network model was subsequently developed and meticulously fine-tuned to evaluate the driver’s level of fatigue by identifying relevant features, including the prolonged duration of eyelid closure, the frequency and occurrence of yawning, as well as the proportion of time during which the eyelids remain closed. The model effectively reduces the misdetection caused by facial expressions and reflects the cumulative nature of fatigue [25]. Gwo-Ching Chang et al. detected the motorist’s instantaneous eye monitoring to assess fatigue driving through an optimized YOLOv7-Tiny model combined with structured pruning and architectural fine-tuning, in which the model storage size was reduced by about 97% and the frame rate was increased by a factor of six, while maintaining high precision and recall [26]. Han Zheng et al. proposed a fatigue detection algorithm adapted to the driver’s facial features using the ShuffleNet V2K16 neural network and the Dlib model to eliminate the problem of poor environmental adaptation in fatigue detection, and achieved 98.8% precision, 90.2% recall, and 94.3% F-Score in real driving scenarios, which significantly improved the detection accuracy and reliability [27]. As deep learning progresses, methods for detecting faces have also undergone significant development.

However, the common low-light environment and special light environment on mountainous roads bring great challenges to the application of face detection technology, and traditional face detection algorithms often fail to accurately detect the driver’s facial features under dark light conditions, thereby impacting the precision of fatigue recognition. To enhance the effectiveness and precision of object detection [28] and fatigue monitoring in this specific environment, this study proposes a YOLOv5 neural network fatigue detection model incorporating a DCN convolution module, a TA mechanism, and a Wingloss function. Through deep reinforcement learning [29,30], the feature detection of face contours [31] and key local regions (eyes, mouth) in complex backgrounds is enhanced, focusing on important information and irrelevant background noise in the image, and the ability to capture small changes in the face is enhanced.

Despite the implementation of policies in 2022 to strictly investigate fatigue driving behaviors, a comparison of traffic accident statistics from 2022 and 2023 reveals no significant reduction in fatigue-related accidents. This indicates that the risks of fatigue driving remain inadequately addressed. Therefore, the core scientific proposition of this study is to improve the YOLOv5 deep learning model by incorporating the unique characteristics of mountainous road environments, using facial feature recognition to enhance the accuracy of detecting driver fatigue. This approach aims to provide a computationally efficient solution that can be deployed on a wider range of hardware, contributing to reducing the dangers associated with fatigue driving.

## 2. Improved Fatigue Detection

### 2.1. Improved Model

**Figure 1:**
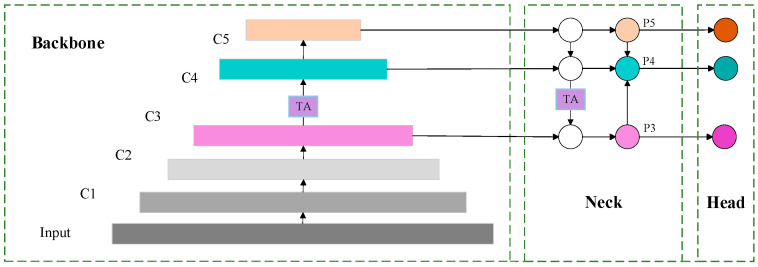
Improved FPN and PANet structures in YOLOv5.

**Figure 2:**
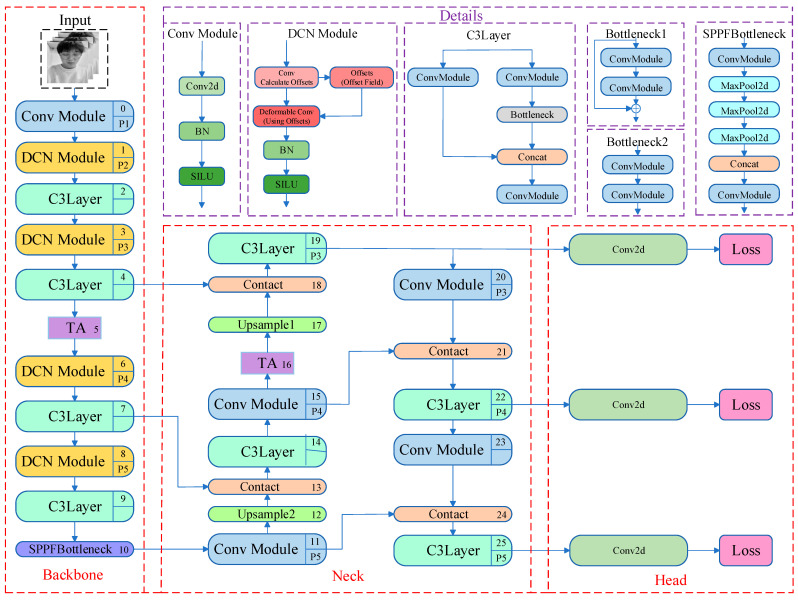
Improvement of YOLOv5 network structure.

The YOLOv5s base model faces certain challenges in face feature detection and driver fatigue determination in complex mountainous road environments. Therefore, a fatigue state detection model for drivers on mountainous roads incorporating DCN convolution, the TA mechanism and Wing loss function is proposed. The improved model utilizes the DCN convolution module to strengthen the adaptability to face expression and gesture changes in complex backgrounds, which enhances the detection accuracy of key local regions (eyes, mouth). Meanwhile, the implementation of the TA mechanism enables the model to concentrate more on the critical details within the image, effectively suppresses the irrelevant background noise, and enhances the overall robustness of the system. Moreover, the heightened responsiveness of the Wing loss function to minor inaccuracies enables the model to detect subtle variations in facial features more precisely. The architecture of the enhanced network model is depicted in Figure 1 and Figure 2.

### 2.2. Improvement of the Backbone Network

Within the YOLOv5 foundational framework, the input image features are extracted through the Conv Module convolution module. The base model is not sufficiently flexible in extracting features of highly deformed targets and targets of varying shapes in dynamic or low-light environments. Therefore, the Conv Module convolution module of the YOLOv5 model is replaced with the DCN convolution module, as indicated by the yellow rectangles in the YOLOv5 network structure in Figure 1.

When the previous layer of the feature map appears, the DCN module covers the pixel area corresponding to the feature map based on the dimensions of the convolution filter; the acquired offset is employed to modify the sampling location of the convolution filter on the feature map; the stride of the convolution filter shift and the padding approach for the feature map borders are regulated by the stride and padding settings; the weight and bias parameters are invoked to complete the feature map related computation; in the end, the altered feature representation is transmitted via the output pathway.

**Figure 3:**
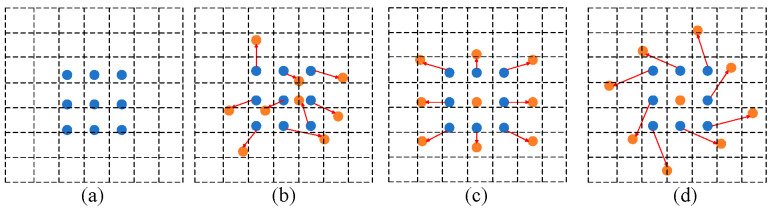
Different adoption points of deformable convolution, including fixed grid (**a**), learned offsets (**b**), adaptive sampling (**c**), and flexible deformation (**d**).

For the DCN convolution principle shown in Figure 3, Figure 3a demonstrates the standard 3 × 3 convolution kernel sampling method, while Figure 3b shows the variation in deformable convolution sampling points adjusted by offsets; Figure 3c,d demonstrate specific examples of deformable convolution. Essentially, deformable filtering depends on shifts derived through network training, and these shifts enable the filter’s sampling locations on the input feature representation to adjust according to the requirements of the target area. In this approach, each sample point is given an offset computed from the feature map through an additional convolutional layer, and these offsets are usually expressed in fractional form, which allows the convolutional operation to focus more precisely on key regions in the image.

**Figure 4:**
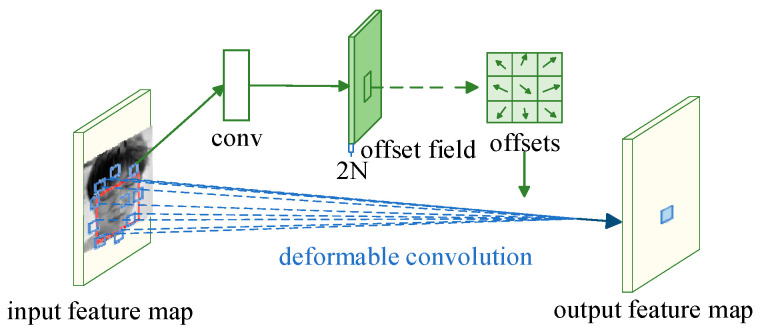
Schematic of deformable convolution.

As illustrated in Figure 4, within the input feature representation, the area sampled by traditional convolution is typically a square zone corresponding to the dimensions of the convolution filter (represented by the red box), while the sampling zone for deformable filtering is achieved through the shifted points depicted by the blue box, which are adjusted according to the learned offsets. This shows the main difference between deformable convolution and traditional convolution, that is, deformable filtering can flexibly modify the sampling locations based on the specific requirements of the features.

(1) $$y(p_{0})=\sum_{p_{n}\in R}w(p_{n})\cdot x(p_{0}+p_{n}+\Delta p_{n})$$

Equation (1) modifies a convolution process expression, where $p_{0}$ represents a location within the feature representation, $p_{n}$ denotes a shifted location of the convolution filter, $\Delta p_{n}$ denotes the offset of the $p_{n}$ position, w($p_{n}$) represents the convolution filter’s weight at location $p_{n}$, and *y*($p_{0}$) is the eigenvalue at the output feature map position $p_{0}$.

**Figure 5:**
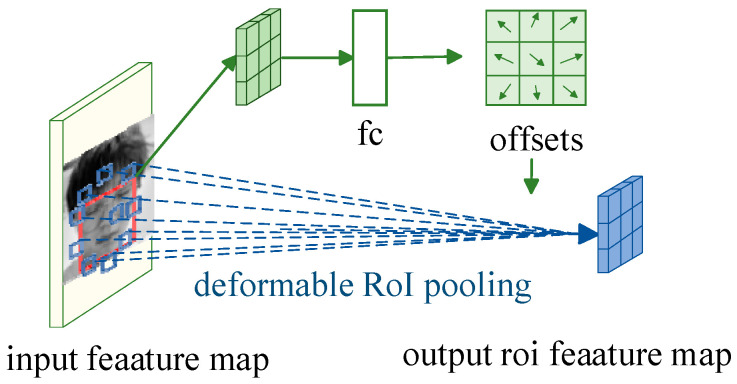
Deformable RoI pooling.

As illustrated in Figure 5, the input picture features are first extracted by a convolutional neural network to form an input feature map, which passes through a fully connected layer (fc), generating a set of offsets (offsets), which are then applied to the spatial locations of the RoI pooling, making the RoI pooling deformable, and finally generating the output feature map.

(2) $$y(i,j)=\sum_{p\in bin(i,j)}x(p_{0}+p+\Delta p_{ij})/n_{ij}$$

Equation (2) is the formula for deformed RoI pooling. After introducing the offset $\Delta p_{ij}$, a new pooling process applies the offset to each mesh. y(i,j) in Equation (2) denotes the output eigenvalue of the lower (i,j) network, *x*($p_{0}+p+\Delta p_{ij}$) denotes the eigenvalue of the input eigenmap at position $p_{0}+p+\Delta p_{ij}$, and $n_{ij}$ is the pixel value in the (i,j) network.

**Figure 6:**
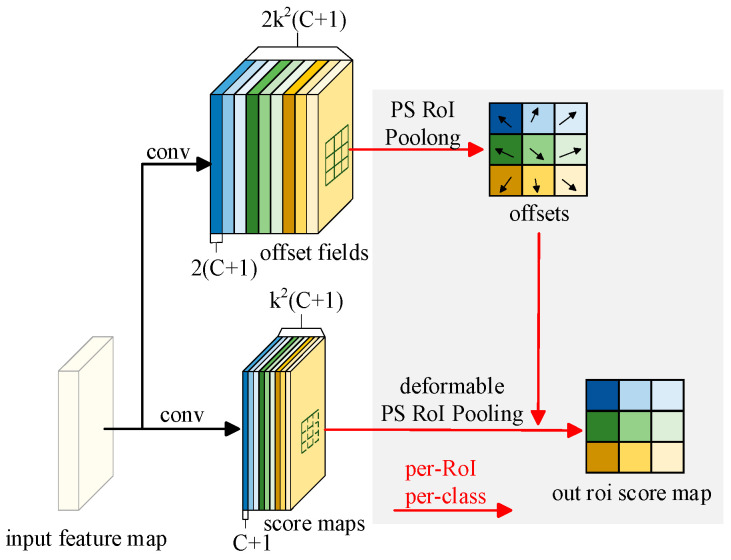
Deformable PS RoI pooling.

The procedure and concept of deformable PS RoI pooling are illustrated in Figure 6. Initially, the incoming image was contrasted for attribute extraction in order to acquire the incoming attribute representation, offset fields were generated through the first convolutional layer, the shape of *2k*^2^(*C* + 1) was used to adjust the spatial position of PS RoI pooling so that it can adapt to the structure of the incoming attribute representation, and following the PS RoI pooling operation, the scored maps were pooled according to the predefined rules. The feature map of the RoI was generated, and the offset of each position was provided by the offset field so that the pooling operation has deformation capability. Through the second volume layer score maps were generated, shape *k*^2^(*C* + 1) was adjusted after deformation PS RoI pooling, and the role of the offset field was considered so that the feature map of each RoI could be adaptively adjusted according to the specific geometry, so as to generate a more accurate RoI feature map.

After introducing the offset, bilinear interpolation is required to obtain the offset pixel points to replace the actual pixel points on the input feature map. Bilinear interpolation means that the weighting weights are set by calculating the distances of the four nearest existing pixel points around the interpolated point, within lateral and vertical axes, and thereafter, the intensity at the interpolated position is determined as the weighted aggregate of those four locations. The intensity levels at the estimated locations are eventually acquired. First, linear interpolation is conducted in the x-direction and is calculated as follows:

(3) $$f(R_{1})\approx \frac{x_{2}-x}{x_{2}-x_{1}}f(Q_{11})+\frac{x-x_{1}}{x_{2}-x_{1}}f(Q_{21}),R_{1}=(x,y_{1})$$

(4) $$f(R_{2})\approx \frac{x_{2}-x}{x_{2}-x_{1}}f(Q_{12})+\frac{x-x_{1}}{x_{2}-x_{1}}f(Q_{22}),R_{2}=(x,y_{2})$$

Linear interpolation from the y-direction is calculated as follows:

(5) $$f(p)\approx \frac{y_{2}-y}{y_{2}-y_{1}}f(R_{1})+\frac{y-y_{1}}{y_{2}-y_{1}}f(R_{2})$$

Combining the results of the two linear interpolations yields the value of point *P* = (*x*,*y*).

(6) $$f(x,y)\approx \frac{f(Q_{11})}{\left(x_{2}-x_{1})(y_{2}-y_{1}\right)}(x_{2}-x)(y_{2}-y)+\frac{f(Q_{21})}{\left(x_{2}-x_{1})(y_{2}-y_{1}\right)}(x-x_{1})(y_{2}-y)+\frac{f(Q_{12})}{\left(x_{2}-x_{1})(y_{2}-y_{1}\right)}(x_{2}-x)(y-y_{1})+\frac{f(Q_{22})}{\left(x_{2}-x_{1})(y_{2}-y_{1}\right)}(x-x_{1})(y-y_{1})$$

**Figure 7:**
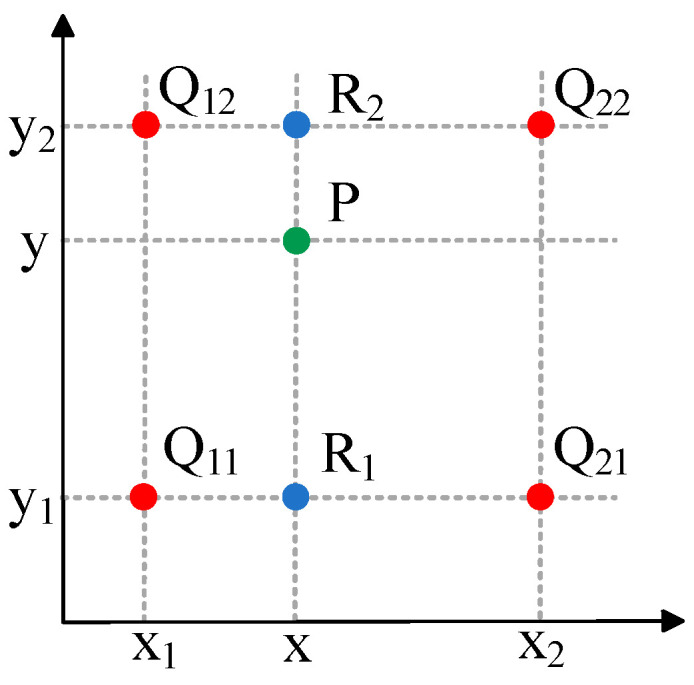
Schematic diagram of bilinear interpolation.

This intensity level at location P is computed from the weighted aggregate of *Q*_11_, *Q*_12_, *Q*_21_, and *Q*_22_, i.e., four points, and the weight of each point is determined by the distance of each point from point, P as shown in Figure 7. For the adaptive convolution component Figure 3 illustrates the structural representation of adaptive convolution. In this structure, the offset is generated by a separate convolutional layer that is specialized in calculating the offset and is not involved in the final convolution operation. In the diagram, N denotes the dimensions belonging to the convolution filter, for instance, a 3 × 3 convolution filter where N equals 9. The green process in the figure represents the process of this dedicated convolutional layer in learning the offset, where the offset field (offset field) has 2N channels, which means that each convolutional kernel position learns the offset in two directions (x and y).

DCN convolution occurs at a location that (output feature representation) aligns with a sampling area of dimensions *K × K* (input feature representation), following the process of flexible convolution. Each convolution sampling point of this *K × K* region has to learn a deviation offset and the offset is expressed in coordinates, so an output has to learn 2 × *K × K* parameters. If we suppose an output is of size *H × W*, a total of 2 × *K × K × H × W* parameters have to be learned.

**Figure 8:**
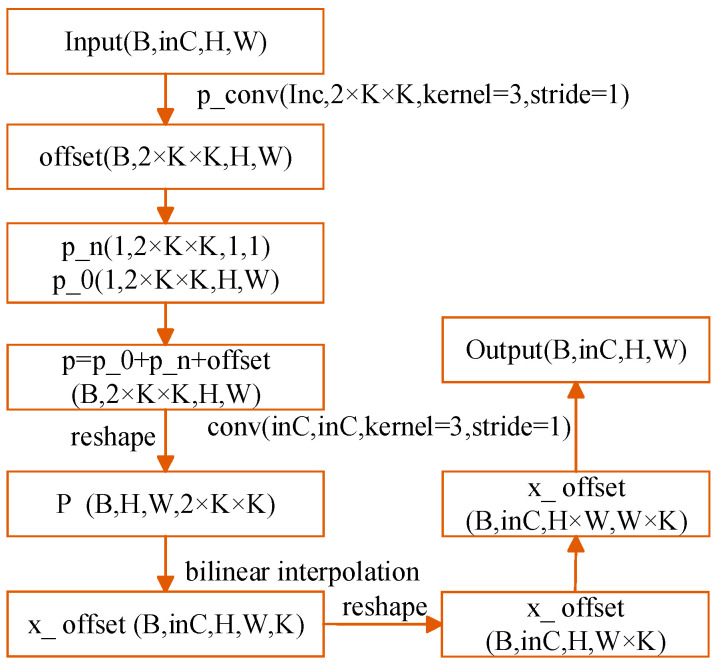
Deformable convolution implementation flow.

That is, the offset field (*N = K × K*) in Figure 8 has the dimension *B* × 2 × *K × K × H × W*, where B stands for Batch_Size. Presuming that the size of the input feature representation measures *B × C × H × W*, all feature representations in a batch (totaling C) utilize one offset field, i.e., each feature map within a batch uses the same offset; flexible convolution does not alter the dimensions of the input feature representation, meaning the output feature representation remains *H × W*.

### 2.3. Incorporate the TA Mechanism

**Figure 9:**
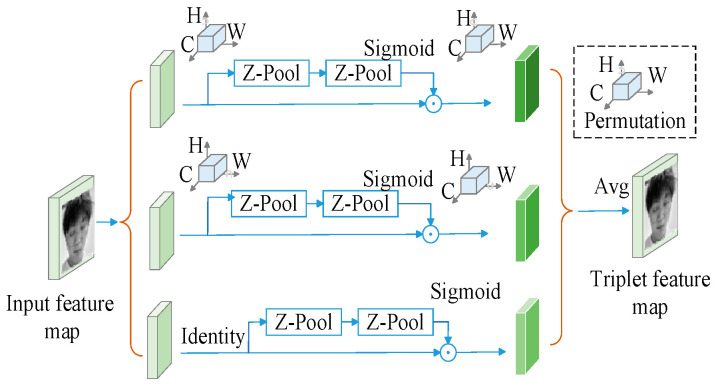
TA mechanism module.

The TA mechanism is added to the backbone and neck networks of YOLOv5, as indicated by the purple rectangles in the YOLOv5 network structure in Figure 2. This mechanism achieves interaction between dimensions through a three-branch structure to calculate attention weights. First, a rotation operation is performed on the input tensor to activate inter-dimensional dependencies. Then, a residual transformation is applied to further strengthen these dependencies, enabling the system to precisely concentrate on key attributes in the image while minimizing unnecessary background interference, its structure is shown in Figure 9.

The Z-pool layer is specifically designed to reduce the dimensionality of a tensor while preserving its rich representation, further reducing the computational burden by combining average pooled features with maximum pooled features. This structural design not only ensures the secure interaction of information, but also optimizes the computational efficiency.

(7) $$Z\text{-}\mathrm{pool}(\chi {)=[\mathrm{MaxPool}}_{0d}(\chi {),\mathrm{AvgPool}}_{0d}(\chi )]$$

**Figure 10:**
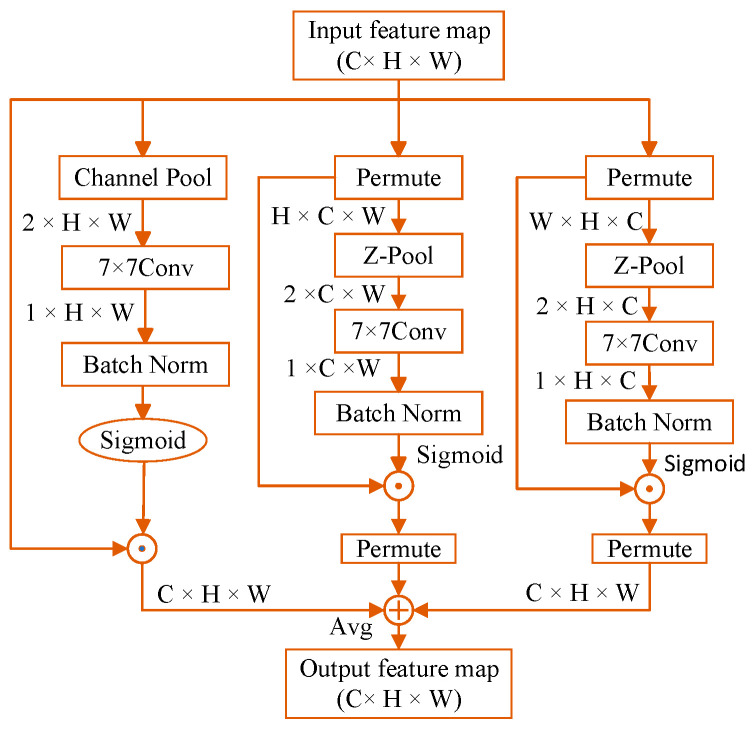
TA mechanism network architecture.

The TA mechanism is a complex network structure that enables effective interactions between different dimensions by applying specific transformations to the input tensor. For an input tensor *χ∈^RC×H×W^*, the TA mechanism comprises three primary branches, each tasked with identifying relationships among various dimensions, as shown in Figure 10.

First, to create a link between the H and C dimensions, the input matrix χ undergoes a 90-degree counterclockwise rotation along the *H*-axis, with ${\hat{\chi }}_{1}$ tensor dimensions as (*W × H × C*). Subsequently, the form of tensor χ is transformed into (2 × *H × C*) through the operation of the Z-Pool layer. This matrix undergoes processing via a *k × k* convolution layer and a batch normalization unit, resulting in an intermediate output of (1 × *H × C*).

Subsequently, the attention coefficients are produced using a sigmoid function, and the final output undergoes a 90-degree clockwise rotation around the *H*-axis, preserving the input tensor’s original dimensions.

During the interaction between the C and W dimensions, the incoming matrix undergoes turned 90 degrees anticlockwise around this *W*-coordinate, and that resulting matrix has dimensions of (*H × C × W*). Through Z-Pool processing, the tensor ${\hat{\chi }}_{2}^{*}$ shape becomes (2 × *C × W*), which is subsequently processed through k × k convolutional and batch normalization layers to produce the intermediate output (1 × *C × W*). Attention coefficients are subsequently produced through sigmoid function activation, and the resulting output restores the initial tensor dimensions using identical procedures.

For interactions between the H and W dimensions, the input tensor *χ*’s channels are compressed from a Z-Pool to two dimensions, resulting in a (2 × *H × W*) tensor. After analysis by the standard convolutional layer and batch normalization, the attention weights of (1 × *H × W*) shape are first formed by the sigmoid function activation layer, and afterward, these coefficients are utilized by the input tensor *χ* to obtain the result ${\hat{\chi }}_{3}$. Finally, the output tensor of the trio of streams is combined through the mean method to form its final output with dimensions (*C × H × W*).

(8) $$y=\frac{1}{3}(\bar{{\hat{\chi }}_{1}\sigma (\psi_{1}({\hat{\chi }}_{1}))}+\bar{{\hat{\chi }}_{2}\sigma (\psi_{2}({\hat{\chi }}_{2}^{*}))}+\chi \sigma (\psi_{3}({\hat{\chi }}_{3}))$$

### 2.4. Introducing the Wing Loss Function

The Wing loss function mainly replaces the bounding box regression part of the YOLOv5 network for application scenarios that require high sensitivity to small errors in face keypoint detection. The core idea of Wingloss is to provide a larger gradient when the error is small, which helps the network to more accurately adjust the predicted location of key points. This loss function consists of two parts: a linear part and a nonlinear part, through which the structure can effectively balance the stability and convergence speed in the learning process. The formula of Wing loss function is shown in Equation (9):

(9) $$\mathrm{Wingloss}(y,\;\hat{y})=\left\{\begin{array}{l} \omega \ln(1+\frac{\left|y-\hat{y}\right|}{\epsilon }),\;\mathrm{if}|y-\hat{y}|<\omega \\ |y-\hat{y}|-C,\;\;\;\;\;\;\mathrm{otherwise}\; \end{array}\right.$$

In this equation, *y* represents the actual value, $\hat{y}$ denotes the estimated value, and *ω* along with *ϵ* are variables governing the curve’s form, and *C* is a constant, computed from ω and *ϵ*, which is used to provide a smooth transition between the two parts of the loss function.

### 2.5. Implementation of YOLOv5

This study adopts YOLOv5 as the base model due to its balance between accuracy and speed, making it suitable for resource-constrained devices. YOLOv5’s customizability allows the integration of high-performance modules, such as deformable convolutions, the TA mechanism, and WingLoss, to enhance facial feature recognition accuracy. While higher versions like YOLOv10 offer improved accuracy, their complexity and higher hardware requirements limit their suitability for real-time applications. With advancements in technology and hardware, we plan to explore the application of newer YOLO versions in future work.

## 3. Fatigue Characterization

### 3.1. Eyes and Mouth Feature Extraction

**Figure 11:**
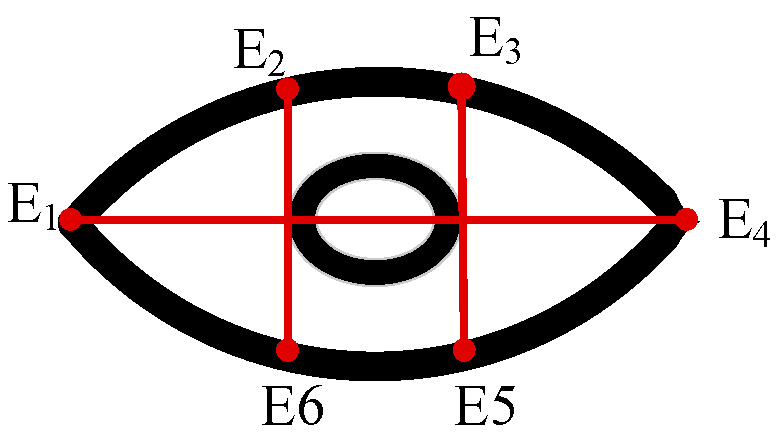
Schematic of Eye Aspect Ratio.

After the face is recognized, the model will mark the key feature points of the eye on the face and then measure the Eye Aspect Ratio (EAR) based on the Euclidean distance [32], which will be used as a criterion to judge the eye fatigue state, as shown in Figure 11. The calculated EAR value is greater than the set state threshold, indicating that the eyes are currently in an open condition. The formula is as follows:

(10) $$EAR=\frac{\left||E_{2}-E6||+||E_{3}-E_{5}|\right|}{2||E_{1}-E_{4}||}$$

**Figure 12:**
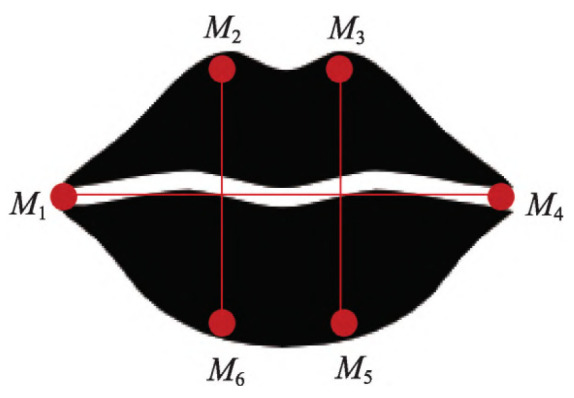
Schematic diagram of the aspect ratio of the mouth.

Following the completion of face detection, the model will label the key feature points of the mouth and then use the Euclidean distance to compute the Mouth Aspect Ratio (MAR) [33], thus acting as a standard for assessing the condition of the mouth, as shown in Figure 12. If the computed MAR value exceeds the predefined threshold, this indicates that the mouth is currently in an open state. The equation is provided below:

(11) $$MAR=\frac{\left||M_{2}-M6||+||M_{3}-M_{5}|\right|}{2||M_{1}-M_{4}||}$$

### 3.2. Determination of Eye and Mouth Fatigue

After numerous experiments and validations, the research team at Carnegie Mellon University proposed an authoritative fatigue detection parameter, PERCLOS (Percentage of Eyelid Closure Over the Pupil Over Time). PERCLOS represents that the proportion belonging to the pupil covered by eyelid closure over a given period of time is one of the most important indicators for assessing fatigue or drowsiness. This indicator is measured by quantifying the duration of the degree of eye closure over a given period of time and is calculated as follows:

(12) $$PERCLOS=\frac{N_{CloseFrame}}{N_{TotalFrame}}\times 100\%$$

**Figure 13:**
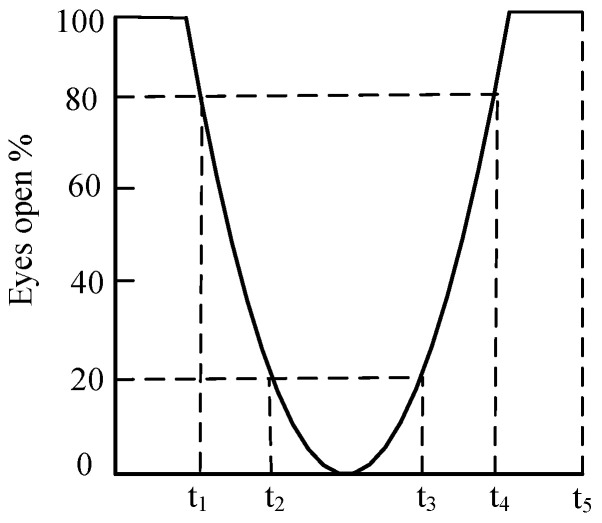
PERCLOS-based eye opening and closing curves.

In Equation (12), *N_CloseFrame_* is the number of frames in which the eye was closed during the given time period, and *N_TotalFrame_* represents the complete count of frames within the identical time interval. The *PERCLOS* method utilizes the criteria EM, P70, and P80 to determine whether the eye is open or closed at that time, as shown in Figure 13. Specifically, a person’s eyes are recognized as being in a closed-eye state based on the corresponding criteria when the degree of eye closure is at or above 50%, 70%, or 80%. Of these criteria, P80 was found to have the strongest correlation with fatigue status, and therefore the P80 criterion was used as a basis for judgment in assessing whether or not a subject was in a state of fatigue.

The number of yawns per unit time was used as a fatigue parameter for the mouth state. If the *P_Yawn_* value exceeds the set threshold, it is determined that the person under test is in a state of fatigue. The fatigue parameter equations for the mouth state are shown below:

(13) $$P_{Yawn}=\frac{N_{Yawn}}{T}\times 100\%$$

### 3.3. Experimental Environment and Evaluation Metrics

The experimental setup includes the following configurations: the operating system is Linux; the CPU is Intel i5-13500HX; the GPU is NVIDIA RTX4060; the system has 16 GB of RAM, and CUDA version 12.1; the development environment is PyCharm; the development framework is PyTorch; and the programming language is Python 3.10. The image size is 640 × 640, with a momentum factor of 0.9, an initial learning rate of 0.001, and the number of training epochs is 200.

Inside the aforementioned formula, *N_Yawn_* represents the count of yawns detected, while T denotes the overall duration. In the present paper, mean accuracy mAP is used as the main evaluation index of the model, and the related basis formula is as follows:

(14) $$P=\frac{TP}{TP+FP}\times 100\%$$

(15) $$R=\frac{TP}{TP+FN}\times 100\%\;$$

(16) $$AP=\int_{0}^{1}P\left(r\right)\;\mathrm{dr}\times 100\%$$

(17) $$mAP=\frac{\sum_{i=1}^{n}AP}{n}\times 100\%$$

In the above formula, *P* represents the precision rate, signifying the ratio of samples predicted as positive; *R* refers to the recall rate, describing the ratio of samples that are truly positive instances. *TP* denotes the correctly classified sample, that is, the target is correctly predicted; *FP* is negative sample identification of a positive sample, that is, target misdetection; *FN* is positive sample identification and negative sample identification, that is, missing target detection; *AP* is the average accuracy of the curve area with accuracy *P* as the *Y* axis; recall rate *R* as the *X* axis; n is the sample type; mAP is the average accuracy *AP* value of multiple types.

The dataset was rigorously selected based on the *PERCLOS* parameter, where the eye state was labeled as closed when the degree of eye closure was greater than or equal to 80%, and vice versa for the open state. In the process of detection and judgment, there may be eye details that are obscured and cannot be detected. The frequency of yawns per time unit is also included as a criterion for assessing fatigue, and the combination of the two fatigue determination methods is used as the final fatigue driving judgment standard.

## 4. Experimental Results

### 4.1. Face Detection Experiment

The dataset used for driver face detection is derived from the publicly available WIDERFACE dataset [34]. The WIDERFACE dataset selected 32,203 images for face annotation, and a total of 393,703 faces were labeled with data, of which each contained an average of 12 faces. Each face is labeled with detailed category information, including expression, illumination, blur, occlusion, and pose.

Considering the unique characteristics of the mountain roads in this experiment, a small number of driver face datasets were created using the LabelImg tool. These datasets effectively simulate face detection during mountain driving and allow a more realistic evaluation of the improved model’s accuracy.

**Figure 14:**
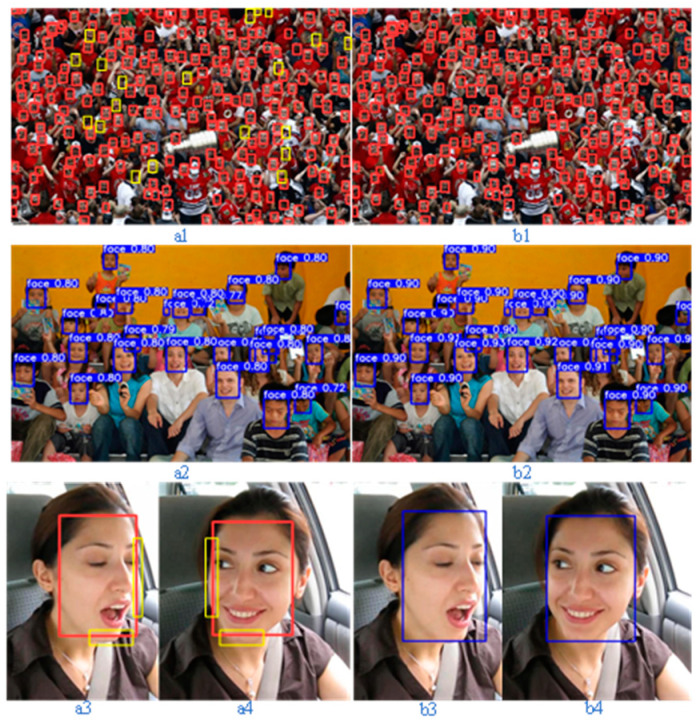
Comparison of face detection before and after model improvement.

To more effectively assess the model’s performance and detection capability prior to (a) and following (b) enhancement, four face images were selected and demonstrated by visualizing the face detection results. Figure 14(a1) shows that the red box is normal detection and the yellow box is missed detection, and there are 20 fewer face detection boxes compared to (b1), indicating that the improved model enhances the detection of occlusion and small targets. Figure 14(a2,b2) shows that the confidence of detecting faces is improved by about 0.1, indicating that the improved model has better confidence loss. Figure 14(a3,a4,b3,b4) shows that the yellow box is the undetected face contour, and the loss of the original model bounding box is greater than that of the improved model, which indicates that the improved model is more adaptable to irregular contours and changes in the face pose.

From Table 1, ShuffleNetv2 and MobileNetv3 are commonly used lightweight networks optimized for resource-constrained devices, utilizing techniques such as grouped convolutions and profundity separable convolutions to achieve a good balance between inference speed and detection accuracy. As shown in Table 1, Model 4 achieves the highest average precision, improving by 1.9% compared to Model 1, while its parameter count increases by 19.7%, reaching the maximum. Models 2 and 3 reduce the parameter count by 50% compared to Model 1, and average precision increases by 1.3% and 1.1%, respectively, but both are still lower than Model 4. The improvement in accuracy is attributed to the characteristics of deformable convolution. Deformable convolution adds an offset to the regular convolution, transforming the conventional rectangular kernel into an irregular shape. This feature allows the kernel to adjust its shape according to the irregularities in facial features (such as the eyes and mouth), focusing on edge features when encountering irregular edges. As a result, it significantly reduces the extraction of irrelevant features while enhancing the focus on edge features, leading to improved model accuracy.

From Table 2, CA and CBAM mechanisms are common attention mechanisms: CA improves the model’s sensitivity to important channels by weighting the information of channel dimensions, while CBAM combines the attention mechanisms of both channel and spatial dimensions to further improve the feature extraction capability. As can be seen from Table 2, the number of parameters of Model 2 and Model 3 increases by 0.3 m and 0.6 m compared with Model 1, and the average accuracy is also improved by 1.1% and 1.3%, whereas the number of parameters of Model 4 increases by 0.7 m to reach 7.8 m, and the average accuracy is also improved by 1.5% to reach a maximum of 78.7% It can therefore be seen that Model 4 meets the requirements of the study better. One of the performance improvements comes from the fact that the TA mechanism is able to focus the model’s attention on important image regions and suppress extraneous background noise through the interactive operation between three different channels: channel, space, and dimension. The mechanism optimizes the flow of image information through rotation and residual transformation, making the model more capable of focusing on facial features in the face of complex background and noise. Thus, the TA mechanism enhances the model’s sensitivity to key facial features in complex backgrounds, thereby improving the accuracy and reliability of the model.

**Figure 15:**
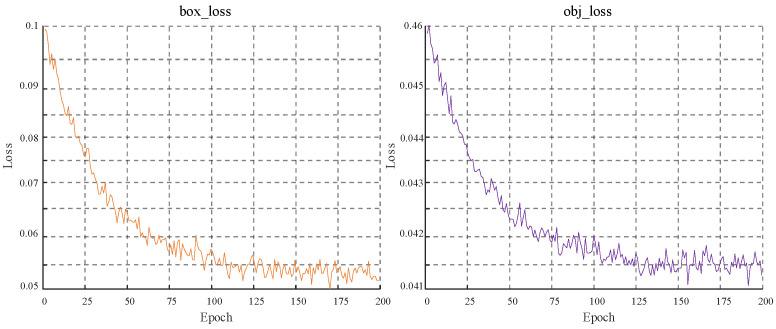
Trend of face bounding box: confidence loss.

As shown in Figure 15, box_loss (bounding box loss) measures the gap between the bounding box and the real bounding box and obj_loss (confidence loss) measures the gap between the predicted target confidence and the real target. As the training progresses, the loss value gradually decreases and eventually stabilizes, indicating that the predicted bounding box is gradually aligned with the real bounding box and the predicted target confidence is getting closer and closer to the real target confidence, showing the improvement of model performance. When the prediction error is small, the Wingloss function uses a linear function to calculate the loss, which gives a large gradient and pushes the model to adjust quickly and accurately on small errors; when the prediction error is large, the Wingloss function uses a nonlinear function to calculate the loss, which reduces the gradient of the large error part and avoids excessive correction in the case of large errors, preventing unnecessary oscillations or over-adjustment.

**Figure 16:**
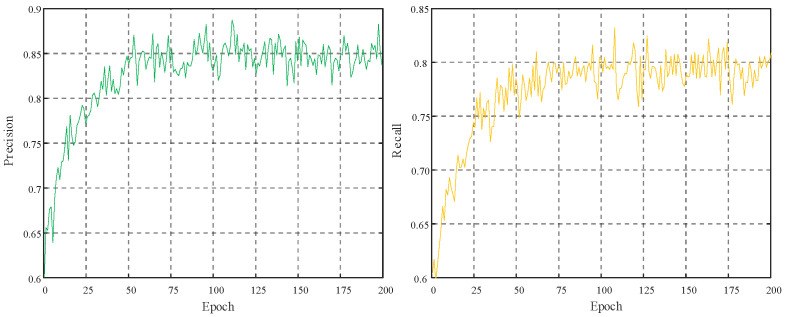
Trend of face detection precision: recall rate.

From Figure 16, it can be observed that the precision and recall are 85% and 79%, respectively indicating that the model achieves good accuracy in predicting positive class samples and recognizing actual positive class samples.

From the experimental results in Table 3, we can see the differences in the number of parameters and mAP (mean accuracy) of the different detection models. The mean accuracy (mAP) of the YOLOv5 base model is 77.2%; the mean accuracy (mAP) of the RetinaFace algorithm model is 85.8%; the mean accuracy (mAP) of the Swin Transformer algorithm model is 85.3%. The average accuracy of the detection of the reformer model increased by 4.9% to 82.1% compared to the YOLOv5 model. From the average accuracy of the latter three models, it can be observed that the gap between the three models is not large, mostly reaching 80% in the actual experiments, and the effect of the gap is minimal. 

**Figure 17:**
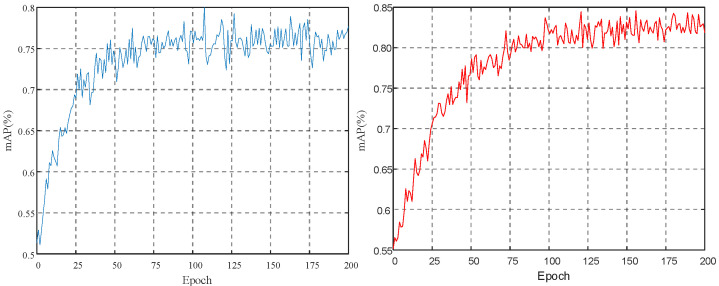
Comparison of accuracy trend before and after model improvement.

As shown by the comparison of accuracy trends in Figure 17, the fluctuation of mAP value of the improved model is smaller, which indicates that the model reduces the risk of overfitting, and reduces the probability of misdetection and omission. From the number of model parameters, it can be seen that the parameters of RetinaFace algorithm and Swin Transformer algorithm are 4 and 5 times larger than the improved YOLOv5 model. Considering the limitations of real-time and hardware arithmetic power and the special characteristics of mountain road environment, the RetinaFace algorithm and Swin Transformer algorithm obviously cannot satisfy the previous two conditions, whereas the improved YOLOv5 model satisfies these three conditions, and the reliability of the practical application is much higher than the other three models.

### 4.2. Ablation Experiment

**Figure 18:**
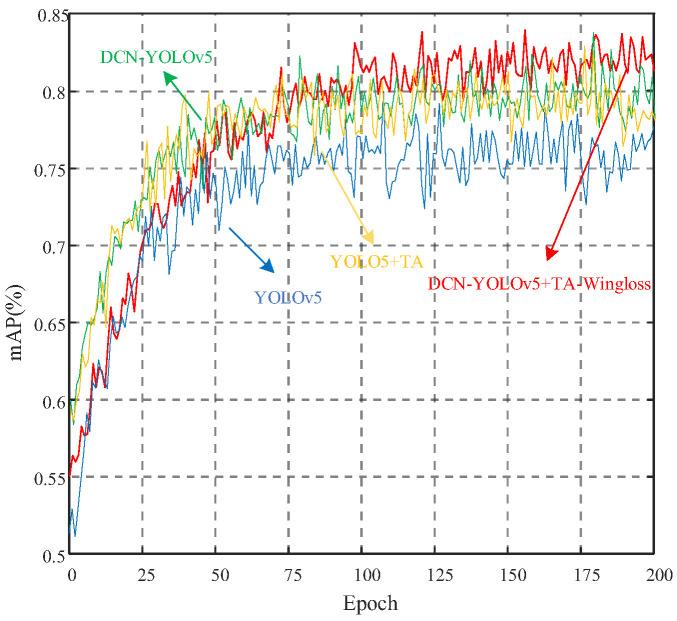
Comparison of ablation experiment accuracy trend.

In order to verify the effectiveness of the improved model, the improved ablation experiments were conducted gradually on the base model, and the results of the ablation experiments are shown in Figure 18. First, replacing the four Conv modules in the YOLOv5 backbone network with DCN modules improved the mAP by about 2% (to 79.1%), which enhanced the model’s adaptation to the changes in the facial features (eyes open, eyes closed, mouth open, and mouth shut) and to low-light scenes. Second, the addition of the TA mechanism alone improved mAP by 1.5% (to 78.7%), enhancing the model’s ability to focus on important facial features in complex backgrounds. Finally, after fusing the DCN, TA, and Wingloss functions, the mAP improves by 4.9% (to 82.1%), which makes the model more accurate in capturing subtle facial features, and performs better in the sensitivity to small errors. Taken together, these improvements effectively enhance the accuracy and robustness of the model, address the shortcomings of the base model in terms of performance, and improve the reliability of fatigue driving detection in different scenarios.

The base model of this study is YOLOv5s and the model itself has a relatively low number of parameters. The last four conventional convolutions are replaced in the backbone network, a TA mechanism is added in the neck network, and part of the loss function is replaced in the head network. The DCN convolution is only 5% more than the number of parameters of the conventional convolution, and the TA mechanism is a highly efficient and lightweight model. The TA mechanism is an efficient lightweight model, and the Wingloss function has similar computational parameters to the original model, and the overall number of parameters in the improved model increases by 30%, and the base is still relatively low. Model computational efficiency and accuracy are less affected by the number of parameters, there is no trade-off, and they do not significantly impact performance.

### 4.3. Driver Fatigue Determination Experiment

The dataset used for fatigue detection is derived from the publicly available YawDD video dataset [35]. The YawDD dataset covers both wearing glasses and not wearing glasses. The dataset simulates four common driving situations, silence, speech, fatigue yawning, and singing. Through the research and investigation of human fatigue state, this experiment takes eye features and yawning fatigue state as the main basis for fatigue judgment. The key point localization algorithm of the face mainly through the regression tree collection maps 68 feature points corresponding to the facial coordinates, and each point is represented by a linear index.

In the picture face feature detection experiment, 300 pictures are extracted by keyframes, and the states of face features (eyes, mouth) in the pictures are labeled with remarks. In the video fatigue detection experiments, 30 awake video samples and 30 fatigue video samples are obtained through editing and processing for driver fatigue state determination.

**Figure 19:**
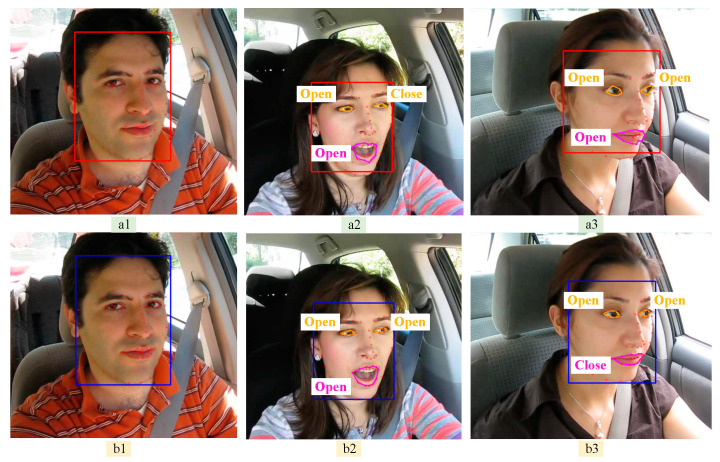
Comparison of eye and mouth state detection before and after model improvement, with the (**a**) series showing the unimproved model and the (**b**) series showing the improved model.

As seen in Figure 19, sequence plot a is before the improvement, and sequence plot b is after the improvement. Among them, the yellow rectangular box area in Figure 19a1 shows 68 facial features in relatively misplaced positions, and Figure 19b1 is the improved normal display effect; while in Figure 19a2,a3, and Figure 19b2,b3, the eye and mouth feature points are connected by curves to display them in a visualized effect. From the figure, it can be seen that there are deviations in the feature points of eyes and mouth as well as deformation of the curve box before the model is not improved, which indicates that the improved model tends to be stable in both face detection and mapping of 68 facial feature points.

**Figure 20:**
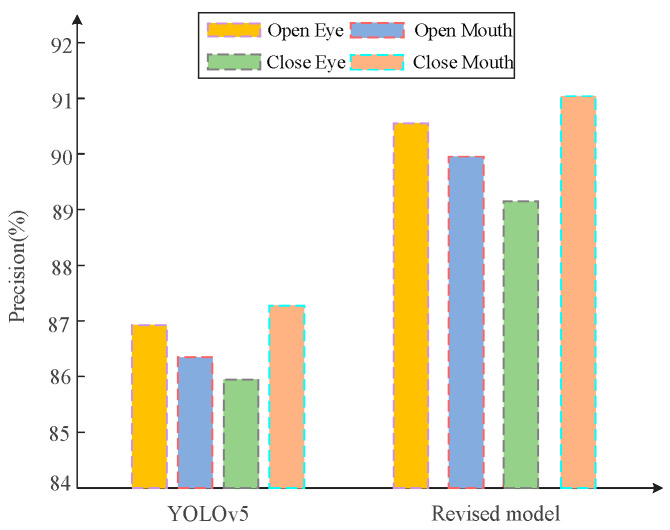
Comparison of eye and mouth state discrimination under different models.

As can be seen from Figure 20, the YOLOv5 base model also presents different accuracy rates due to different feature states when discriminating between the four states of the eyes and mouth, where shutting is the highest and closing the eyes is the lowest, while the average accuracy rate is about 86%. The improved model also presents this kind of situation, which is brought about by the differences in features, and the average accuracy of the improved model is about 90%, which is a corresponding improvement in performance and reliability compared to the base model.

In addition, the driver’s state recognition is not entirely limited by the defects of the model, in which a too dark environment and continuous bumps and jerky body conditions can directly affect the video quality, which in turn affects the detection of faces and the mapping of key points, leading to incorrect state recognition. Also, in some scenarios, due to the individual differences in the driver’s facial features, there may be cases where normal speech and blinking and nodding movements are very close to the set judgment criteria, leading to misjudgment of the normal state.

**Figure 21:**
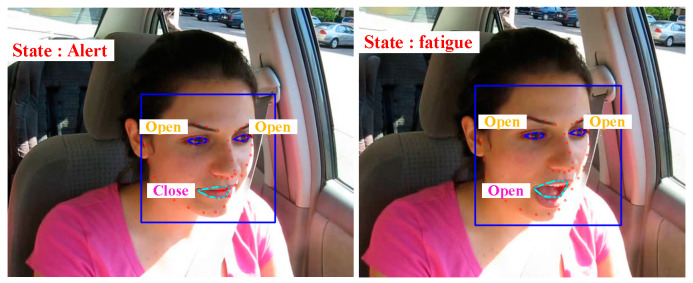
Driver fatigue detection effect.

In real-world scenarios, driver fatigue detection uses real-time video, unlike static image detection. Video provides dynamic information, allowing the model to capture facial expressions, eye changes, and other behaviors for a more accurate fatigue assessment, as shown in Figure 21. Additionally, video offers more information than a single image, aiding in better fatigue detection and early warning.

The clip-processed YawDD dataset is used to assess the model’s effectiveness. This dataset has been labeled with the mental state of the subject under those videos. The detection outcomes are presented in Table 4, where 25 of the 30 samples in the awake state were determined correctly, with an accuracy of 83.3%, and 26 of the 30 samples in the fatigue state were determined correctly, with an accuracy of 86.7%, and the average accuracy of the improved fatigue determination model in this paper was 85%.

## 5. Conclusions

The aim of this study was to explore the problem of driver fatigue state determination in mountainous road environments. Factors such as low light and tree branch shadows, which are unique to mountainous areas, can have an effect on the driver’s face and increase the noise of the video or image, which in turn poses challenges to face detection and fatigue state recognition. To cope with these difficulties, a fatigue detection model combining a DCN convolution module, a TA mechanism, and a Wingloss function is proposed. The DCN convolution module enhances the adaptive ability to face pose changes and face feature curves in complex backgrounds, while the TA mechanism helps the model to focus on the key information in the image, thus ignoring irrelevant background noise and improving the robustness. The Wingloss function makes the model more sensitive to small errors and improves the ability to localize key points. Experiments were conducted using the WIDERFACE dataset and the YawDD dataset to train and validate the model face detection and fatigue determination, respectively, using the *PERCLOS* value of the eye state and the yawning state as the basis for fatigue determination. The experimental results show that the model performs well in fatigue driving state recognition. The research is in the experimental stage. Although it combines the effect of special environments on the recognition of the state to improve, it is not fully equipped with hardware equipment for use in real driving scenarios and there are still some shortcomings, such as its inability guarantee specific performance in different dynamic real environments, such as bad weather or bad road conditions. This is an element that cannot be ignored in future research, and model turnover and hardware equipment upgrades need to be considered in order to achieve better breakthroughs in real dynamic environments. Future research will be devoted to different real dynamic environments, to establish and collect more comprehensive datasets, to collect the influencing elements of state recognition in real scenarios, to optimize the methods and approaches of face detection and feature extraction, to find more accurate judgment criteria from dynamic environments, to improve the methods of state recognition, and to ensure that the proposed system is more adaptive and scalable, which will help reduce the hazards associated with fatigue driving.

## Figures and Tables

**Table 1:** Comparison of different convolutional models.

Model	Parameters/(10^6^)	mAP/%
YOLOv5 (1)	7.1	77.2
ShuffleNetv2-YOLOv5 (2)	3.8	78.5
MobileNetv3-YOLOv5 (3)	3.5	78.3
DCN-YOLOv5 (4)	8.5	79.1

**Table 2:** Comparison of different attention mechanism models.

Model	Parameters/(10^6^)	mAP/%
YOLOv5 (1)	7.1	77.2
YOLOv5+CA (2)	7.4	78.1
YOLOv5+CBAM (3)	7.7	78.4
YOLOv5+TA (4)	7.8	78.7

**Table 3:** Results of face experiments.

Model	Parameters/(10^6^)	mAP/%
Yolov5	7.1	77.2
RetinaFace	38.0	85.8
Swin Transformer	49.6	85.3
DCN-yolov5+TA+Wing loss	9.2	82.1

**Table 4:** Results of face experiments.

State	Videos	Correct Detection	P/%
Alertness	30	25	83.3
Fatigue	30	26	86.7

## Data Availability

The original contributions presented in this study are included in the article. Further inquiries can be directed to the corresponding authors. This study includes the following types of data and images: Images of the Authors: Certain images featured in the manuscript were provided by the authors of the article. The authors confirm their explicit consent for these images to be published as part of the article. Images from Publicly Available Datasets: Additional images in the article were derived from publicly available datasets, specifically: WIDER FACE: A publicly available dataset for face detection research. YawDD: A publicly available dataset for yawning detection research. These datasets are open-access and were used under their respective licensing agreements, which confirm their ethical compliance and legal availability for academic research. No new data collection or direct interaction with individuals was performed for this study.

## References

[1] Amodio A., Ermidoro M., Maggi D., Formentin S., Savaresi S.M. (2019). Automatic detection of driver impairment based on pupillary light reflex. IEEE Trans. Intell. Transp. Syst..

[2] Zhang Z., Ning H., Zhou F. (2022). A systematic survey of driving fatigue monitoring. IEEE Trans. Intell. Transp. Syst..

[3] Sikander G., Anwar S. (2019). Driver fatigue detection systems: A review. IEEE Trans. Intell. Transp. Syst..

[4] Min J.L., Wang P., Hu J.F. (2017). Driver fatigue detection through multiple entropy fusion analysis in an EEG-based system. PLoS ONE.

[5] Zheng W.-L., Gao K., Li G., Liu W., Liu C., Liu J.Q., Wang G., Lu B.-L. (2020). Vigilance estimation using a wearable EOG device in real driving environment. IEEE Trans. Intell. Transp. Syst..

[6] Zhu M., Chen J., Li H., Liang F., Han L., Zhang Z. (2021). Vehicle driver drowsiness detection method using wearable EEG based on convolutional neural network. Neural Comput. Appl..

[7] Arefnezhad S., Samiee S., Eichberger A., Frühwirth M., Kaufmann C., Klotz E. (2020). Applying deep neural networks for multi-level classification of driver drowsiness using vehicle-based measures. Expert Syst. Appl..

[8] Jeon Y., Kim B., Baek Y. (2021). Ensemble CNN to detect drowsy driving with in-vehicle sensor data. Sensors.

[9] Zhao G., He Y., Yang H., Tao Y. (2022). Research on fatigue detection based on visual features. IET Image Process..

[10] Liu M.-Z., Xu X., Hu J., Jiang Q.N. (2022). Real-time detection of driver fatigue based on CNN-LSTM. IET Image Process..

[11] Sheykhivand S., Yousefi Rezaii T., Meshgini S., Makoui S., Farzamnia A. (2022). Developing a deep neural network for driver fatigue detection using EEG signals based on compressed sensing. Sustainability.

[12] Ye M., Zhang W., Cao P., Liu K. (2021). Driver fatigue detection based on residual channel attention network and head pose estimation. Appl. Sci..

[13] Liu Z., Peng Y., Hu W. (2020). Driver fatigue detection based on deeply learned facial expression representation. J. Vis. Commun. Image Represent..

[14] Mu Z., Hu J., Min J. (2017). Driver fatigue detection system using electroencephalography signals based on combined entropy features. Appl. Sci..

[15] Lee J., Lee H., Shin M. (2021). Driving stress detection using multimodal convolutional neural networks with nonlinear representation of short-term physiological signals. Sensors.

[16] Li Z.J., Li S.E., Li R., Cheng B., Shi J. (2017). Online detection of driver fatigue using steering wheel angles for real driving conditions. Sensors.

[17] Li Z.J., Chen L.K., Peng J., Wu Y. (2017). Automatic detection of driver fatigue using driving operation information for transportation safety. Sensors.

[18] Ardabili S.Z., Bahmani S., Zare Lahijan L., Khaleghi N., Sheykhivand S., Danishvar S. (2024). A novel approach for automatic detection of driver fatigue using EEG signals based on graph convolutional networks. Sensors.

[19] Friedrichs F., Yang B. Drowsiness monitoring by steering and lane data-based features under real driving conditions. Proceedings of the 18th European Signal Processing Conference.

[20] Zhao J., Liu Y., Wang X., Zhang Z., Li C., Zhang H. (2023). Driver Fatigue Detection Based on EEG Signals Using a Hybrid Deep Learning Model. IEEE Trans. Intell. Transp. Syst..

[21] Fu S., Yang Z., Ma Y., Li Z., Xu L., Zhou H. (2024). Advancements in the intelligent detection of driver fatigue and distraction: A comprehensive review. Appl. Sci..

[22] Singh A., Kaur J. Driver fatigue detection using machine vision approach. Proceedings of the 3rd IEEE International Advanced Computing Conference (I-ACC).

[23] Li Z., Chen L., Nie L., Yang S.X. (2022). A novel learning model of driver fatigue features representation for steering wheel angle. IEEE Trans. Veh. Technol..

[24] Deng J., Guo J., Ververas E., Kotsia I., Zafeiriou S. RetinaFace: Single-shot multi-level face localisation in the wild. Proceedings of the IEEE/CVF Conference on Computer Vision and Pattern Recognition (CVPR).

[25] Chen J., Yan M., Zhu F., Xu J., Li H., Sun X. (2022). Fatigue driving detection method based on combination of BP neural network and time cumulative effect. Sensors.

[26] Chang G.-C., Zeng B.-H., Lin S.-C. (2024). Efficient eye state detection for driver fatigue monitoring using optimized YOLOv7-Tiny. Appl. Sci..

[27] Zheng H., Wang Y., Liu X. (2023). Adaptive driver face feature fatigue detection algorithm research. Appl. Sci..

[28] Wang D., Yang S.X. (2022). Intelligent feature extraction, data fusion and detection of concrete bridge cracks: Current development and challenges. Intell. Robot..

[29] Chazhoor A.A.P., Ho E.S.L., Gao B., Woo W.L. (2022). Deep transfer learning benchmark for plastic waste classification. Intell. Robot..

[30] Zhang E., Jiang T., Duan J. (2024). A multi-stage feature aggregation and structure awareness network for concrete bridge crack detection. Sensors.

[31] Wang Y., Zhang L., Yao Y., Fu Y. (2022). How to trust unlabeled data? Instance credibility inference for few-shot learning. IEEE Trans. Pattern Anal. Mach. Intell..

[32] Soukupová T., Cech J. (2018). Real-time eye blink detection with application in driver fatigue monitoring. Neurocomputing.

[33] Yao L., Jiang J., Xie L., Bai J., Shi P., Xie Z., Zhou Y. (2021). Driving distraction detection using a hybrid feature model based on multi-scale CNN. IEEE Access.

[34] Yang S., Luo P., Loy C.C., Tang X. WIDER FACE: A Face Detection Benchmark. Proceedings of the IEEE Conference on Computer Vision and Pattern Recognition (CVPR).

[35] Abtahi S., Omidyeganeh M., Shirmohammadi S., Hariri B. YawDD: A Yawning Detection Dataset. Proceedings of the 5th ACM Multimedia Systems Conference (MMSys ’14).

